# Remedies for Sleeplessness

**Published:** 1877-08

**Authors:** 


Remedies for Sleeplessness.—In a recent exchange, Fothergill, after discussion of the cause of sleeplessness, tabulates, as follows, the remedies which have been hitherto the most highly recommended for this complaint:

1. Opium is indicated when sleeplessness is caused by pain; when irritation of the vascular system is present, aconite and antimony are to be combined with it.

2. Hyoscyamus is of service when sleeplessness depends on disease of the kidney.

3. Chloral hydrate is inefficacious in sleeplessness dependent on pain, though it is a hypnotic *par excellence* in the sleeplersness of fever, particularly in children. This remedy is injurious in ill humor, brain exhaustion, and in the sleeplessness of melanchly.

4. Bromide of potassium acts as a sedative either on the brain cells or vessels of the brain; it is indicated in those cases where peripheral irritations are present, and is very beneficial in the sleeplessness which is the result of maladies of the pelvic organs.

5. Alcohol is a powerful hypnotic in those cases in which sleeplessness comes from sorrow, ill humor, and mental disturbances.—*Nash. Jour. Med. and Surg.*

